# Advancing precision cancer immunotherapy drug development, administration, and response prediction with AI-enabled Raman spectroscopy

**DOI:** 10.3389/fimmu.2024.1520860

**Published:** 2025-01-09

**Authors:** Jay Chadokiya, Kai Chang, Saurabh Sharma, Jack Hu, Jennie R. Lill, Jennifer Dionne, Amanda Kirane

**Affiliations:** ^1^ Department of Surgery, Stanford School of Medicine, Stanford University Medical Center, Stanford, CA, United States; ^2^ Department of Electrical Engineering, Stanford University, Stanford, CA, United States; ^3^ Pumpkinseed Technologies, Palo Alto, CA, United States; ^4^ Genentech, South San Francisco, CA, United States; ^5^ Department of Materials Science and Engineering, Stanford University, Stanford, CA, United States; ^6^ Department of Radiology, Molecular Imaging Program at Stanford (MIPS), Stanford University School of Medicine, Stanford, CA, United States

**Keywords:** Raman spectroscopy, label-free analysis, immunotherapy, time analysis, multiomics

## Abstract

Molecular characterization of tumors is essential to identify predictive biomarkers that inform treatment decisions and improve precision immunotherapy development and administration. However, challenges such as the heterogeneity of tumors and patient responses, limited efficacy of current biomarkers, and the predominant reliance on single-omics data, have hindered advances in accurately predicting treatment outcomes. Standard therapy generally applies a “one size fits all” approach, which not only provides ineffective or limited responses, but also an increased risk of off-target toxicities and acceleration of resistance mechanisms or adverse effects. As the development of emerging multi- and spatial-omics platforms continues to evolve, an effective tumor assessment platform providing utility in a clinical setting should i) enable high-throughput and robust screening in a variety of biological matrices, ii) provide in-depth information resolved with single to subcellular precision, and iii) improve accessibility in economical point-of-care settings. In this perspective, we explore the application of label-free Raman spectroscopy as a tumor profiling tool for precision immunotherapy. We examine how Raman spectroscopy’s non-invasive, label-free approach can deepen our understanding of intricate inter- and intra-cellular interactions within the tumor-immune microenvironment. Furthermore, we discuss the analytical advances in Raman spectroscopy, highlighting its evolution to be utilized as a single “Raman-omics” approach. Lastly, we highlight the translational potential of Raman for its integration in clinical practice for safe and precise patient-centric immunotherapy.

## 1 Introduction

The immune system plays a vital role in detecting cancer by recognizing neoantigens generated by tumor cells that can initiate immune responses (1). However, tumors have evolved several strategies to evade immune detection (2). These include downregulating antigen presentation, which impairs the ability of immune cells to recognize and attack tumor cells, and expression of surface protein ligands, such as Programmed Death-Ligand 1 (PD-L1), that interact with immune checkpoint proteins, such as Programmed Death Protein 1 (PD-1), on immune cells (3). Tumor-secreted factors modulate the tumor immune microenvironment through several mechanisms, including: i) releasing immunosuppressive cytokines such as IL-2, TGF-β, IL-10, IL-35 and VEGF, which inhibit various immune cell activities (4); ii) releasing tumor-derived exosomes which contain immunosuppressive molecules, including TRAIL, Fas-L, PGE-2, etc (5); and iii) recruiting regulatory immune cells such as regulatory-T cells, tumor associated macrophages, and myeloid-derived suppressor cells to the tumor site (6). Epigenetic modulation within cancer cells can also silence genes related to antigen presentation (7). To effectively deploy immunotherapy, it is essential to accurately detect and classify the evasion tactics of cancer cells. Our manuscript discusses how Raman spectroscopy, as a label-free, reliable, and cost-effective technology, can sense these tactics across the immunological synapse.

Various immunotherapy strategies currently utilized include immune checkpoint inhibitors (ICIs), cancer vaccines, adoptive cellular therapies (ACT), cytokines, targeted antibodies including T cell-engaging bispecifics, and adjuvants & immunostimulants. Although these approaches have led to improved outcomes for some patients, their benefits are often limited to a small and unpredictable segment of cancer patients. This has led to increased cases of immune-related adverse events (irAEs) (8, 9). For example, in melanoma, where ICIs are the mainstay treatment, the overall response rate is only 30-45% for the most common single-agent anti-PD-1 approach (10). Further, many cancers, such as pancreatic adenocarcinoma, have unique biologic environments such as high levels of fibrosis, contributing to immune cell resistance and evasion that render these immunotherapeutic agents significantly less effective (11, 12). Therefore, accurately assessing a patient’s tumor microenvironment (TiME) and predicting their response to immunotherapy are essential for maximizing treatment effectiveness. An important step towards this is precise biomarker prediction which helps in establishing more accurate, individualized profiles to guide immunotherapeutic selection (13, 14). As many existing biomarker predictive models rely on single-omics data, which may not capture the complex biological interactions involved in tumor immunology, their predictive power has been limited (15, 16). Multi-omics approaches that combine genomic (17), transcriptomic (18), proteomic (19) lipidomic, and metabolomic data can improve the accuracy of response predictions (20–23). In a recent study, Kong et al. utilized a machine learning framework that integrated various -omics data to predict responses to ICIs in melanoma, gastric cancer, and bladder cancer, demonstrating superior predictive capabilities compared to traditional biomarkers. Investigators curated data from more than 700 ICI-Treated patients’ samples with clinical outcomes and transcriptomic data. Their network-based ML algorithm showed significantly better performance in predicting ICI treatment responses in all the above-mentioned types of cancers compared to existing models, demonstrating network biology as a powerful means to identify robust biomarkers (16).

Multi-omics technologies have increased our understanding of the complex inter- and intra- molecular cross-talk between immune cells and tumor cells within TiME. However, working with large analytical and statistical datasets generated by single or spatial technologies presents significant computational hurdles (24). One major issue is the batch effects caused by using different analytical techniques employed in -omics data collection (25). These techniques are costly, time consuming, and require extensive labeling steps which may require disruption of native biological environments for the cells of focus (26, 27). Raman spectroscopy can effectively harmonize all the -omics techniques for analyzing TiME interplay and its intricate changes under a single platform. Additionally, a combination of Raman and traditional multi-omics can also leverage the strengths of both methodologies, including the high sensitivity, multiplexing capabilities, rapid analysis, and non-destructive, label-free nature of Raman, alongside the specificity and extensive data provided by traditional -omics approaches. In the past decade, label-free Raman spectroscopy has found significant applications in cancer diagnostics, particularly in cell type differentiation (28–30) and metabolite characterization (31–33). It also allows for the identification of biochemical changes within tumors, enhancing our ability to monitor responses to therapies more efficiently (34). These studies provide the foundation for deploying Raman spectroscopy as a platform for immunotherapy development, administration, and response monitoring.

In this perspective, we discuss the principles and role of Raman spectroscopy in immunotherapy. In section 2, we describe advances in nanophotonics which render Raman suitable for non-invasive, label-free detection of the TiME at the single-cell to few-molecule level. We also discuss the role of machine learning and artificial intelligence (ML/AI) in Raman spectral analysis and data interpretation. Section 3 describes the role of Raman spectroscopy in identifying, characterizing, and analyzing the complex inter- and intra- metabolic and phenotypic changes occurring within TiME, as well as Raman spectrosocpy’s role in predicting responses to various immunotherapeutic treatments. Section 4 outlines the current analytical advancements in Raman spectroscopy within the field of immunology. Finally, Section 5 explores how Raman spectroscopy can serve as a unifying, multi-omic technique that stitches genomic, transcriptomic, proteomic, and metabolomic data, as well as a potentially low-cost tool with translational potential in clinical settings.

## 2 Nanophotonic-enhanced Raman spectroscopy and AI-enabled interpretation

**Figure 1:**
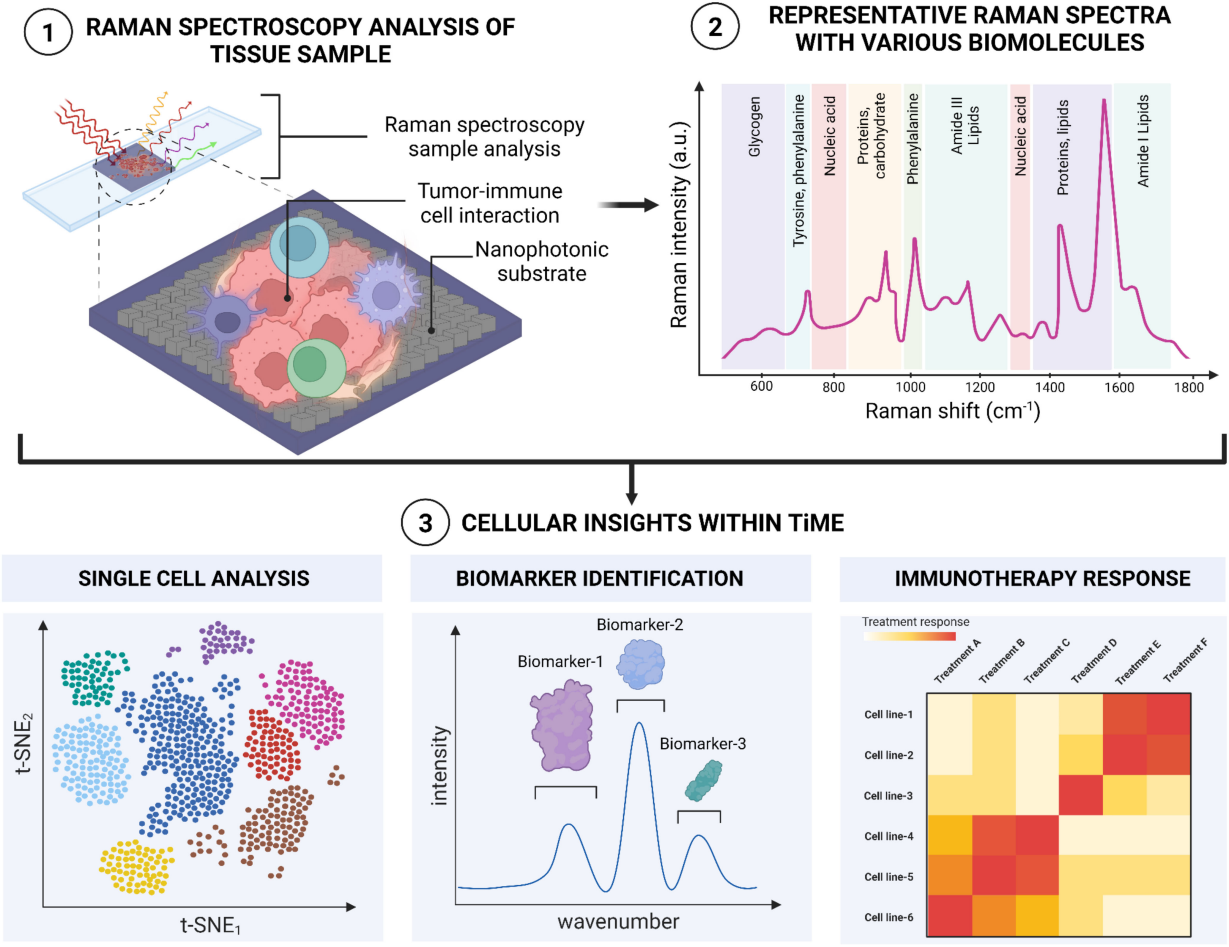
A schematic illustration of Raman spectroscopic workflow for analyzing and observing inter and intra cellular interactions within TiME.

Raman spectroscopy (RS) is a non-invasive, vibrational spectroscopic method that examines the composition, structure, and vibrational energy states of materials (including molecules and cells). In RS, a sample is illuminated with monochromatic light. When the incident light interacts with molecular vibrations in the sample, photons can be inelastically scattered and re-emitted with either lower or higher energy (**Figure 1**). This energy difference, known as a Raman shift, provides a distinct molecular “fingerprint” of the material (35). By analyzing the unique spectral fingerprints of molecules fundamental in cellular biology, RS can provide detailed insight into the molecular composition and the structural and functional makeup of cells and tissues, both *in vivo* and *ex vivo (* 36, 37). For example, there are biologically-relevant windows (38, 39) that elucidate biomarkers spanning lipids (40, 41), proteins and peptides (42, 43), metabolites (44–46) and nucleic acids (47, 48) (**Figure 1**). In turn, these markers can demarcate normal and malignant cells (49, 50) and stratify cancer types (51) or pathologic grades (52, 53), facilitating potential early diagnosis and intervention pathways. As a non-destructive optical technique, Raman spectroscopy can be seamlessly integrated with other modalities on the same sample, allowing for multi-omic resolution in a single measurement.

Although Raman spectroscopy is non-invasive and highly specific in providing molecular and structural information, a major challenge of spontaneous RS lies in its intrinsically weak scattering process. Because of the low likelihood of a Raman scattering event [roughly 1 in 10E6-7 incident photons (54–56)], complementary strategies have been adopted to address its signal intensity and enhance sensitivity. The emergence in the fields of nanophotonic materials and machine learning models, in particular, have improved Raman sensitivity and resolution and to enable deeper spectral interpretation.

One strategy to amplify the signal-to-noise ratio of Raman is through surface-enhanced Raman scattering (SERS), which uses optically resonant surfaces or nanoparticles (NPs) to increase the Raman cross-section (**Figure 1**). Vast literature has been published using metallic nanostructures for SERS. When light interacts with these metallic nanostructures, the electrons in the metal oscillate in resonating manner, creating an intensified electromagnetic field known as a plasmon resonance on the surface. This additional field strength localization intensifies the light interaction that occurs between molecules, with enhancement coefficients ranging from 10^4^-10^8^, and as high as 10^11^ (45–47). The resulting process generates highly-detailed, vibrational spectra, making it particularly useful in fields like cancer immunotherapy (48, 49), biochemistry (50, 51), medical diagnosis (52), and surgical treatments (53). SERS studies employing colloidal NPs have shown extensive success in cancer biological interrogation, from Liu et al. exploiting Au/Ag nanostar geometries to quantify BRAF gene mutations in colorectal cancer with comparable LOD to qPCR, to Sun et al. leveraging Au nanorods as a multifunctional agent to identify and induce photothermal ablation of tumor margins (45, 46). Recent advances in large-area nanoarray fabrication leveraging self-assembled NP aggregation or nanolithography have led to the rise and potential of SERS-active substrates. Zhao et al. designed one such substrate by fabricating nanoarrays of plasmonic trimers to successfully label adenocarcinoma, squamous carcinoma and benign tumor samples across fresh lung tissues (57). These SERS-active devices can yield comparable enhancements to colloidal NPs, all while improving sample adhesion and hotspot uniformity and distribution.

Although there is less literature, recent innovations in dielectric-based substrates for SERS present an advantageous opportunity for material and biological characterization. Unlike metallic nanostructures, which exhibit high photothermal effects damaging cells or altering biomolecular structures, dielectric nanostructures undergo minimal heat conversion, making them highly suitable for biological preservation and measurement reliability (58, 59). Advancements in highly resonant, high quality-factor (Q) metasurfaces have also overcome conventionally limited electromagnetic field enhancements (60–62), yielding Raman scattering efficiencies comparable to plasmonic counterparts (58, 63, 64). In work by Cambiasso et al. and Romano et al., for example, dielectric nanodimers and photonic crystals were utilized to demonstrate Raman spectral amplification across β-carotenal monolayers and Raman analytes with minimal absorption loss (65, 66). Silicon-based designs, in particular, can further leverage the device footprint scaling of matured CMOS infrastructure (67). Barkey et al. demonstrated one such design by pixelating 2D arrays of Si-ellipse pairs to resolve real-time conformational dynamics of photoswitchable lipid membranes representative of cell membrane behavior (68). These large-area fabricated arrays can enable homogenous SERS regions for rapid spatial profiling all while providing compatibility to assess the same sample with other modalities.

Enhancing the utility of Raman spectroscopy can be achieved by incorporating machine learning (ML) and artificial intelligence (AI), which can extract underlying spectral features linked to biological and chemical responses. Spectral information from RS is often feature-rich, but the unprocessed information can be complex and noisy. As a result, employment of both more traditional statistical approaches and newer deep learning algorithms can be utilized to isolate pertinent information from background and extract insights in an otherwise opaque spectra. Dimension reduction techniques adopted prior to analysis can improve feature selection, reduce overfitting, and improve computational runtime, all while preserving original spectra information. Linear techniques such as principal component analysis (PCA) can decompose large feature sets into smaller ones encapsulating the most significant spectral patterns and differentiators, while nonlinear reduction methods like t-distributed stochastic neighbor embeddings (t-SNE) or uniform manifold approximation (UMAP) can help contextualize the local and global structural relationship of Raman spectra datasets. Classification algorithms can further intake the Raman spectra and provide distinct cell type labeling to predict post-treatment outcomes in untested samples. Support vector machines (SVMs) and Random decision forests (RFs) can be used to robustly classify cancer subtypes as recently demonstrated in brain tissue (69, 70) and in breast cancer garnering an accuracy of +97% (69, 70). Advances in multilayer architectures such as convolutional neural networks (CNN) and residual neural networks (ResNet) have further increased the predictive capacity of RS, even against high inter-patient variability and complex background sources. For example, in melanoma, where the clinical diagnostic sensitivity and specificity ranges from 40-80%, the implementation of artificial neural networks on Raman spectra resulted in an improved sensitivity and specificity of 85% and 99%, respectively (53). Such integration of deep learning and the continued advancements in AI can stand to provide a powerful opportunity to analyze Raman spectra beyond single cells and across the tissue domain. Further, as discussed later in the perspective, integration of RS with existing multi-omics and spatial-omics data, using existing AI models, could offer a more comprehensive understanding of tumor heterogeneity.

## 3 Role of Raman spectroscopy in characterizing tumor-immune microenvironment

**Figure 2:**
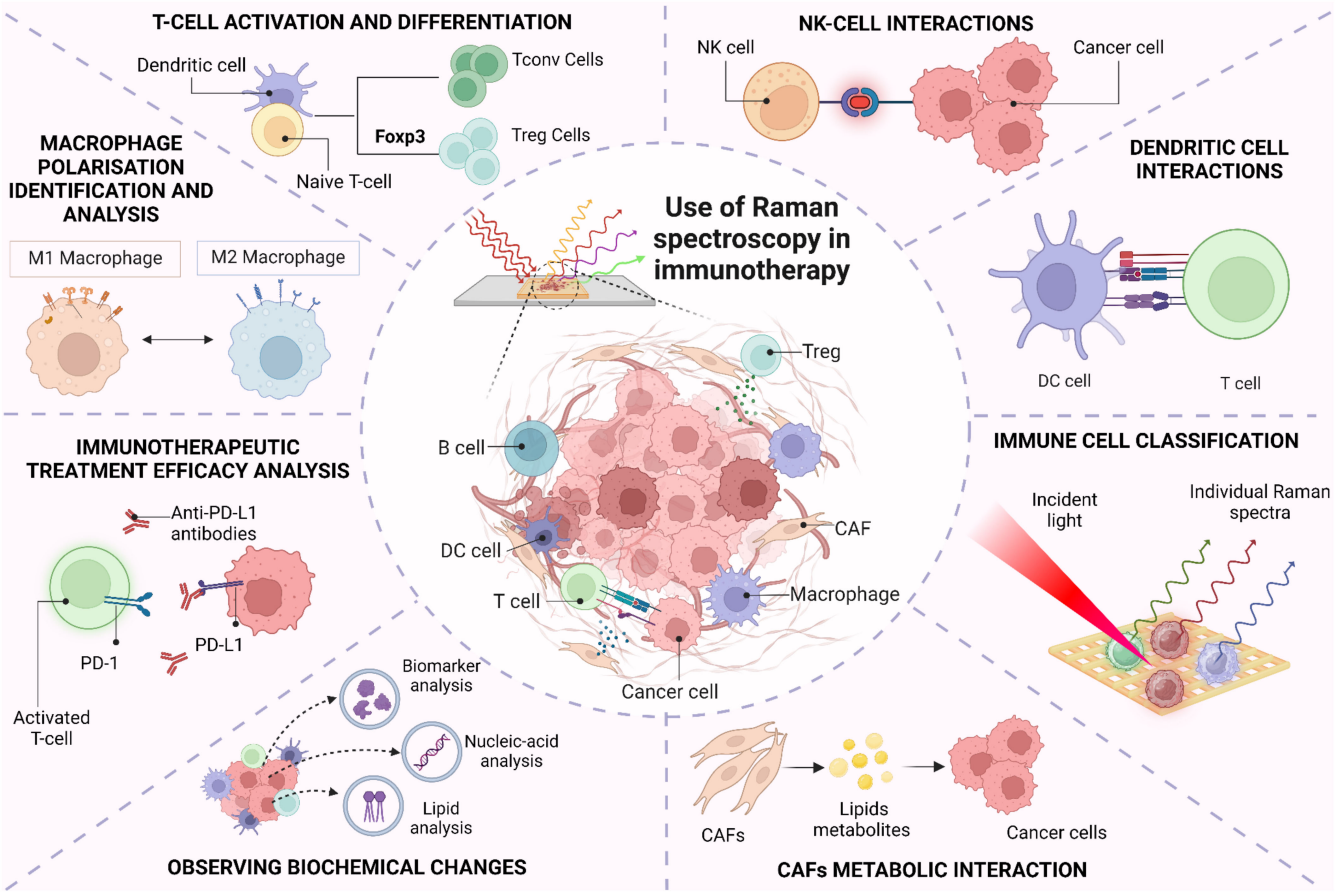
Use of Raman spectroscopy in immune cell classification and its interaction within tumor-immune microenvironment.

The TiME is a complex and diverse ecosystem containing a variety of immunosuppressive cells, including tumor cells, cancer-associated fibroblasts (CAFs), vascular endothelial cells, suppressive myeloid cells, regulatory T (Treg) cells, and regulatory B cells. Increasing evidence strongly suggests that TiME plays a significant role in immune checkpoint inhibitors’ responses, tumor immune surveillance, and immunological evasion (71, 72). Paidi et al. showed evidence that label-free Raman spectroscopy can show TiME compositional changes in response to ICIs. Using CT26 murine colorectal tumor xenografts, they compared tumor responses with treatment across three doses of anti–CTLA4 and anti–PD-L1 antibodies each. They determined that ICI exposure significantly changes the composition of the TiME independent of conventional cellular, molecular, or proteomic characterizations (34). This ability to assess multiple biomolecular changes simultaneously adds significant depth in understanding the TiME and response to therapies. **Figure 2** highlights the multitude of signals that Raman spectroscopy can provide about the TiME. As seen, Raman spectroscopy can be used in differentiating various cancer and immune cell types, including B cells, cytotoxic T cells, helper T cells, NK cells, and dendritic cells. For instance, Chen et al. employed Raman spectroscopy to accurately identify various subsets of immune cells, including T-lymphocytes, dendritic cells, and natural killer (NK) cells, distinguishing CD56+ NK cells from CD4+ and CD8+ T cells with specificities reaching 93% and 96%, respectively. The differentiation between CD4+ and CD8+ T cells was less effective, yielding a specificity of 68% and a sensitivity of 69%, suggesting that these closely related cell types present more challenges in their identification (73). Conventional techniques for immune cell identification and complex classification of the TiME currently relies on extensive labeling for label-based techniques, due to the need to both “rule-in” and “rule-out” broad cell surface markers and utilize multiple labels related to functional behavior and activation status. The exploration of RS to distinguish cell types has been provocative, here we highlight several critical cell types that have been shown to be highly distinguishable by RS (73). While the Raman spectra of these immune cells may appear quite similar, data analysis techniques can reveal the subtle distinctions among them (73–75). Raman spectra can also provide information about the activation states of these cells, including macrophage polarization and T-cell state responses (eg, from activated to exhausted.) Single-cell Raman analysis can further reveal how different cell types interact within the TiME. Finally, Raman can help elucidate tumor heterogeneity and how the spatial structure of the tumor impacts immune responses, currently a major obstacle for effective immunotherapy (76). In this section, we will explore the utility of RS in characterizing, classifying and analyzing different inter- and intra-molecular interactions between immune cells within the TiME.

### 3.1 Macrophages

Macrophages, essential phagocytic and antigen-presenting cells, exhibit a diverse functional spectrum from immunosuppressive, tumor-promoting behaviors to highly inflammatory responses. Their role in the tumor microenvironment is pivotal, as they can either support tumor control or contribute to autoimmune toxicities. Conventionally, differential expression levels of surface polarization markers, such as CD11b, CD80, CD54, CD163 and CD206, are used to differentiate macrophage phenotypes, however the transition from inflammatory to immunosuppressive behavior is highly linked to metabolic switching that can be detected by Raman spectroscopy. In a study by Naumann et al., distinct features of monocyte-derived macrophages, including naïve M0, classically activated M1, and alternatively activated M2 phenotypes were detected by analyzing 65 chemically fixed primary human monocyte-derived macrophages from three donors in combination with N-FINDR spectral unmixing. The authors identified polarization-dependent spectral features associated with the chemical composition of lipids, proteins, and nucleic acids across macrophage phenotypes. Pro-inflammatory M1 macrophages displayed a significantly higher lipid content compared to M0 and M2 phenotypes. M2 macrophages exhibited reduced triacylglycerol content but increased fatty acids. These spectral distinctions facilitated the development of models for automated classification of M1 macrophages, achieving a classification accuracy of 86%, with a sensitivity of 93% and specificity of 85% (77). In another study by Lu et al., macrophage response to biomaterial implants was examined to gain insights into the immune system’s foreign body reaction. Two types of macro-encapsulation pouches (PVDF and TPU-chronoflex) were implanted in streptozotocin-induced diabetic rat models for 15 days. Their research demonstrated that label-free Raman microspectroscopy could effectively identify extracellular matrix (ECM) components within the fibrotic capsule and distinguish between pro-inflammatory M1 and anti-inflammatory M2 macrophage activation states. Significant spectral changes in the nuclei of M1 and M2 macrophages indicated variations in nucleic acid methylation, a key process in fibrosis progression. Specifically, increased peak intensities at 857 cm^−1^ and 879 cm^−1^ in M2 macrophages were linked to proline, hydroxyproline, tryptophan, and tyrosine, suggesting that M2 macrophages have lower methylation levels than M1 macrophages (78). Thus, RS plays an important role in analyzing biochemical changes in lipids, proteins, and nucleic acids across macrophage phenotypes and identifies extracellular matrix (ECM) components.

### 3.2 T-lymphocytes

**Figure 3:**
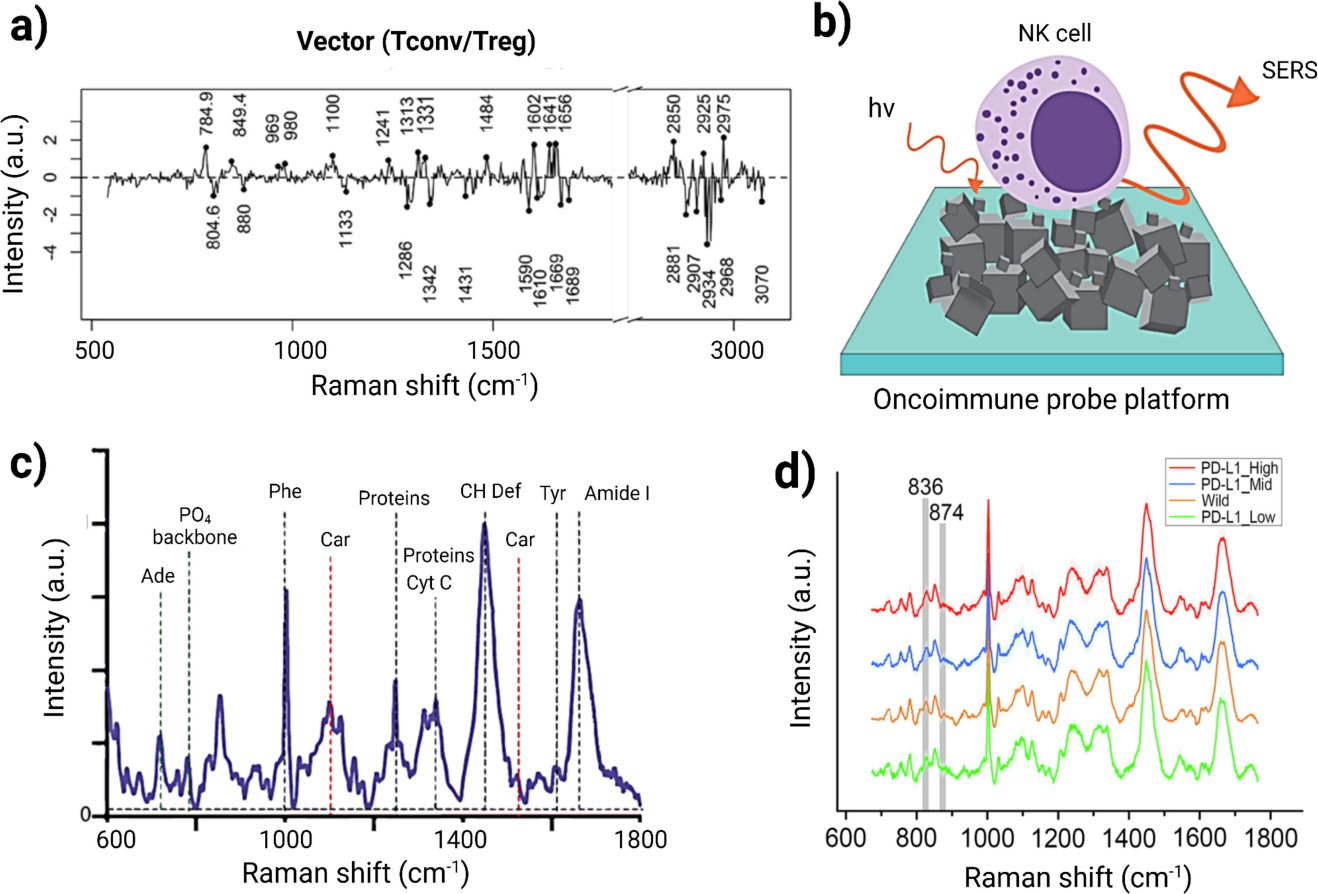
**(A)** Classification efficacy for human Tconv/Treg cells utilizing a separation vector to detect human Treg cells. Adapted with permission under a Creative Commons CC-BY License from ref (79). **(B)** Schematic illustration of NK cells on the OncoImmune probe platform, synthesized with 3D networks of nickel- nickel oxide nanocubiforms. **(C)** Representative Raman spectra of NK cells illustrating the presence of several biomolecules within NK cells. Adapted with permission under a Creative Commons CC-BY License from ref (80). **(D)** Average Raman spectra for PD-L1 expression in cancer cells. Adapted with permission under a Creative Commons CC-BY License from ref (81).

T cells are important effector cells in the TiME, including cytotoxic and regulatory subtypes that attack cancers or suppress immune responses to cancers, respectively. T cell classifications, like macrophages, generally require multiple labels, such as CD3, CD4, and CD8, to define subtype in addition to a multitude of co-stimulatory signals, such as activating ligands or regulating checkpoints to modulate the degree of amplification for T cell responses. Authors Pavillon et al. leveraged the non-tissue destructive nature of RS to monitor live T cell development *in vitro*, demonstrating that without directly describing the cell surface features of these traditional labels, other nuanced molecular changes related to cell state development and activation had high correlation with the transition points identified by label-based assays (29). The sensitivity in this assay also successfully delineated between activation and differentiation by detecting differences in the *in vitro* stimulated cells versus ex vivo activated T cells that otherwise would have required multiple additional labeling steps to define naive versus effector cells. Regulatory T cells (Tregs) are crucial for maintaining immunological self-tolerance and have been identified as having an important role in immunotherapeutic failures. The findings by Pavillon et al. indicated that Raman could distinguish Treg subpopulations without altering cell integrity (29) by the detection of intracellular transcription factor Foxp3, a specific Treg marker. Since Foxp3 is not detectable in live cells, the authors employed RS to reliably identify and isolate functional Treg populations. They sorted conventional T (Tconv) and Treg cells using FACS with Foxp3-hCD2 surface staining, followed by Raman measurements on the isolated populations. A ML model was then developed to differentiate between Tconv and Treg cells, achieving an accuracy of 78.3% on test data, comparable between models trained on naive cells and those based on FACS-sorted data (78.25% for FACS vs. 77.9% for naive cells). When they applied confident learning (CL) to filter out samples with low-probability labels, the model achieved a remarkable 92% accuracy. **Figure 3A** illustrates the classification of human Tconv/Treg using the CL model transformation. Here, negative bands observed can be linked to specific protein structures, such as the amide III α-helix (at 1340 cm^−1^ and 1286 cm^−1^) and amide I (at 1619 cm^−1^ and 1669 cm^−1^. Conversely, the primary positive bands appear to be associated with DNA/RNA, indicated by cytosine/uracil rings indicated at 785 cm^−1^. This approach also enabled the distinction of human Tconv and Tregs from PBMCs with similar accuracy despite donor variability. However, a notable limitation of this method is its throughput; the automated sequential detection system currently processes approximately 1,000 cells per hour, which is insufficient for applications requiring millions of cells (79).

### 3.3 Natural Killer cells

Natural Killer (NK) cells are lymphocytes that play a crucial role in targeting viruses and cancer cells, particularly cancer stem cells (CSCs), which are linked to therapeutic resistance and tumor relapse (82, 83). Ishwar et al. explored the profiling of circulating NK cells as a diagnostic tool using SERS-driven liquid biopsy. The authors specifically synthesized an OncoImmune probe platform to detect metabolic changes in NK cells when they interact with tumor cells, illustrated in **Figure 3B**. Raman spectra of tumor-free NK cells exhibited characteristic bands associated with carbohydrates, proteins, and lipids, including peaks at 1450 cm^−1^ (CH deformation), 1661 cm^−1^ (amide I), 1555 cm^−1^ (amide II), and 1337 cm^−1^ (amide III) (**Figure 3C**). In contrast, tumor-associated NK cells showed altered spectral intensities, indicating an active response to tumor recognition. A decrease in the peak at 520 cm^−1^ suggested changes in Killer Immunoglobulin Receptor (KIR) expression due to CSC interaction. PCA revealed distinct clustering of NK cell signatures associated with breast, lung, and colon CSCs compared to non-cancer-associated NK cells. Utilizing machine learning, the study demonstrated that features of NK cell activity could accurately identify cancer from non-cancer samples using just 5 µL of peripheral blood, achieving 100% accuracy for cancer detection and 93% for localization. This research also highlights the importance of material advances for amplifying the SERS signal, where hybrid material consisting of nickel and nickel oxide produced an enhanced and reproducible SERS signal. This marker-free method generated a detailed NK cell metabolic profile that could be highly advantageous for cellular diagnostic applications. Thus, label-free SERS technique can be used for profiling immune cells and their metabolic changes in difficult to detect tumors such as small-cell lung cancer, triple-negative breast cancer, and colorectal adenocarcinoma (80).

### 3.4 Dendritic cell interactions

Dendritic cells (DCs) play a crucial role in cancer immunotherapy by interacting with cancer cells and presenting tumor antigens to T cells. When DCs capture antigens from cancer cells, their maturation status determines the immune response. Fully mature DCs effectively present these antigens on major histocompatibility complex (MHC) molecules, activating both CD4+ helper and CD8+ cytotoxic T cells. Enhancing DC function and antigen presentation is a key strategy in developing effective cancer immunotherapies (84). T cell receptors (TCRs) form an immunological synapse (IS) with antigen–MHC complexes and co-stimulatory ligands on dendritic cells (DCs), characterized by a distinct “bull’s-eye” structure known as the supramolecular activation cluster (SMAC). Zoladek et al. employed label-free confocal Raman micro-spectroscopy (CRMS) to analyze the IS formed between laminin-treated DCs and T cells *in vitro*. They compared Raman spectral images with immunofluorescence imaging to identify signatures of key macromolecules, including nucleic acids, lipids, and proteins. Using a 785 nm laser, the study assessed the impact of laminin treatment on the DC–T cell junction by capturing images of control and treated DCs stained with phalloidin. Laminin treatment enhanced actin filament polarization and improved IS formation at the DC–T cell interface. The Raman spectra revealed detailed actin distribution in the IS, with characteristic peaks at 1450 cm−1 (CH deformation), 1661 cm^−1^ (amide I), 1555 cm^−1^ (amide II), and 1337 cm^−1^ (amide III). A significant band at 1003 cm^-1^ correlated to histone proteins present in the nucleus. For both DC and T cells, Raman spectral images in the 788 cm^-1^ band exhibit good concordance with the DAPI image, demonstrating the potential of CRMS for non-invasive imaging of live immune cell interactions and providing insights into the dynamics of the immunological synapse (85). This research plays an important role in designing dendritic cell based immunotherapies by providing real time data regarding DC-T cell interactions within TiME.

### 3.5 Cancer-associated fibroblasts

Cancer-associated fibroblasts (CAFs) are integral to the tumor microenvironment, often contributing to immunosuppression by stromal remodeling that protects cancer cells or communication with multiple immune cells via secreted factors. CAFs undergo metabolic changes that aid in tumor growth through interactions with cancer and stromal cells, their inherent plasticity leads to dynamic shifts in the fibroblast population. This emphasizes the need for precise evaluation of CAF’s phenotypic and functional heterogeneity (86). Lipid metabolites released by CAFs not only facilitate metastasis but also serve as indicators of aggressive cancer types (87). The accumulation of lipids within the tumor microenvironment provides fatty acids to nearby tumor cells, fueling their energy needs. Since obesity is characterized by high levels of fatty acid, its impact on CAF’s lipid metabolism remains poorly understood. Yeu et al. investigated this relationship using Raman spectroscopy as a non-invasive technique to analyze lipid metabolite changes in CAFs from endometrial cancer (EC) patients having different BMI. The study focused on Raman spectral regions associated with lipid biochemical changes (600–1800 cm^–1^ and 2800–3200 cm^–1^). Through direct band and ratiometric analyses, researchers observed slight shifts in the CH2 symmetric stretch of lipids at 2879 cm–1 and CH3 asymmetric stretching from proteins at 2932 cm^–1^ in overweight or obese patient CAFs compared to non-obese patients. These shifts indicated a higher lipid content and increased lipid saturation in the obese CAFs and, with the help of PCA, metabolic phenotypes linked to obesity and cancer progression were effectively differentiated. The identification of specific Raman spectral signatures in CAFs offers valuable insights into the tumor microenvironment’s influence on EC progression (88).

### 3.6 Tumor-immune microenvironment biomarker prediction

Designing effective studies to evaluate immunotherapeutic treatment efficacy poses a significant challenge, particularly regarding immune cell interactions and its characterization. The interactions within the TiME are intricate and dynamic, and understanding these interactions are essential towards predicting immunotherapy response. For instance, in Merkel cell carcinoma (MCC), research has shown that tumor-associated macrophages (TAMs) can express immunosuppressive markers that inhibit T-cell function. TAMs exhibit an immunosuppressive gene profile typical of monocytic MSDCs and notably express several immune checkpoint molecules that are potential therapeutic targets, such as PD-L1 and LILRB receptors (89, 90), which are absent on tumor cells. A study analyzing 54 tumor samples prior to immunotherapy revealed that a specific subset of TAMs (characterized by CD163+, S100A8+, CD14+) preferentially infiltrate tumors with a higher presence of CD8+ T-cells. Furthermore, a higher density of these TAMs was linked to resistance against PD-1 blockade therapies (91). In another study, single-cell RNA sequencing (scRNA-seq) revealed that a lower immune-cell infiltration (CD8 T-cell, NK cells, and a complete absence of γδ T-cells) was more common in acral melanoma when compared to non-acral melanoma (92). Tumor heterogeneity not only affects initial responses but also contributes to acquired resistance to immunotherapies which takes the form of immunosuppression and antigen escape. As tumors undergo immunotherapeutic treatments, they may develop subpopulations of cells that are resistant to immune-mediated cell death (76). These cases have been noted in melanoma (93) and breast cancer (94) studies and highlight the necessity of characterizing immune cell subsets and their activation states to tailor immunotherapy approaches effectively.

Raman spectroscopy has shown to be effective in immunological whole-tumor profiling, with Ou et al. showing the simultaneous detection of PET and SERS in monitoring the dynamics of tumor cell compositions *in vivo*. Currently, PD-L1 expression in TiME is the most important clinical biomarker assessed prior to immunotherapy use. High levels of PD-L1 have been associated with better outcomes in various cancers, including melanoma (95), lung cancer (96), and metastatic renal cell carcinoma (97). However, due to tumor heterogeneity, the relationship between PD-L1 expression levels in tissues and therapeutic responses to anti-PD-1/PD-L1 treatments is not always consistent (3, 98). This variability can be partially attributed to the influence of N-linked glycosylation on PD-L1, which may hinder the binding of commonly used anti-PD-L1 antibodies, thus the rapid glycosylation assessment possible with RS could enhance the reliability of PD-L1 as a biomarker for predicting responses to immune checkpoint therapies (99). Additionally, the expression of PD-L1 in both tumor and immune cells has been correlated to ICI clinical responses, making accurate PD-L1 characterization a valuable companion diagnostic for PD-1/PD-L1 inhibitors.

To assess PD-L1 expression, Zhou et al. developed an intra-operative technique using label-free Raman spectroscopy combined with ML for data analysis and visualizing PD-L1 in glioma cells, macrophages, CD8+ T cells, and normal cells. They employed stainless steel and Calcium Fluoride substrates to minimize background signals. Principal component analysis (PCA) was first utilized to differentiate Raman spectra between PD-L1_G_ (high PD-L1 expression in glioma cells) and PD-L1_L_ (Low PD-L1 expression in glioma cells) subgroups. Random Forest (RF) analysis identified five significant peaks at 723, 783, 837, 874, and 1437 cm^−1^. PD-L1_G_ exhibited stronger intensities at 837, 874, and 1437 cm^−1^ compared to PD-L1_L_, which showed weaker intensities at 724 and 783 cm^−1^. **Figure 3D** represents the average Raman spectra for PD-L1 expression in cancer cells. The peak intensities at 837 cm^-1^ and 834 cm^-1^ showed a positive linear correlation with PD-L1 levels. This is correlated with the increased expression levels of PD-L1 in glioma cells. The study also explored spectral differences among PD-L1_G_, PD-L1_T_ (high PD-L1 expression in T-cell), and PD-L1_M_ (High PD-L1 expression in macrophage) subgroups, revealing biological correlations between cell types and their Raman spectral features. Notably, ganglioside, phosphatidylcholine (PC), and cytochrome-c contributed to PD-L1_T_, while sphingomyelin and oleic acid were linked to PD-L1_M_. The relationship between spectral features and biomolecule levels were qualitatively assessed across different cell types. Multiple ML algorithms—including CLS, HCA, SVM, and SA—were employed to analyze Raman spectra for model training and visualize PD-L1 expression in the glioblastoma immune microenvironment. This method for detecting the PD-L1 biomarker can be extended to other tumor biomarkers or target cells of interest, enhancing intra-operative diagnostics for surgical guidance and post-operative immunotherapy (81).

### 3.7 Predicting response to immunotherapeutic treatment

The current clinical metrics for prediction and evaluation of response to anti–CTLA4 and anti–PD-L1 immune checkpoint inhibitors (ICIs) in the TiME are not very effective (100, 101). PD-L1 score of 0, for example, can still demonstrate response to therapy and score is not currently utilized as a selection criteria for therapy (102). A liquid biopsy strategy combining blood count parameters, clinical characteristics, and serum lactate dehydrogenase predicted the response of patients without metastatic disease to anti–PD-1 therapy with about 60% accuracy (103). Studies have also leveraged PD-1/PD-L1 and CTLA4– targeting antibodies radiolabeled with 89Z for evaluating the tumor uptake of therapeutics using PET imaging; however, such measurements are associated with challenges (104). To address the challenges in predicting immunotherapy responses, Paidi et al. employed label-free Raman spectroscopy to monitor compositional changes in the tumor immune microenvironment (TiME). Using a CT26 murine model of colorectal cancer, tumors were treated with anti–CTLA-4 or anti–PD-L1 antibodies. Snap-frozen tumors were thawed, flattened, and positioned between a quartz coverslip and an aluminum block with PBS to prevent dehydration, with the quartz selected for its low fluorescence interference. The team utilized a fiber-optic probe connected to a portable Raman system (830 nm diode laser) on a motorized translational stage to gather data. They collected 7,500 spectra from 25 tumors over a 5-second acquisition time. Ex vivo Raman mapping conducted three days post-treatment yielded 7,585 spectra from approximately 300 spatially distinct points across the tumors. Key Raman peaks identified included 849 cm⁻¹ (C–O–C skeletal mode of polysaccharides), 1,260 cm⁻¹ (amide III of proteins), 1,301 cm⁻¹ (lipid and collagen bending), 1,448 cm⁻¹ (lipid and collagen CH₂ bending), and 1,657 cm⁻¹ (amide I of proteins). Comparisons between treatment groups revealed subtle yet statistically significant differences in lipid, nucleic acid, and collagen value, suggesting that responses to anti–CTLA-4 and anti–PD-L1 therapies influence TiME composition (34). These findings align with emerging research on the role of metabolism and the tumor microenvironment in shaping immune responses. Variations in lipid-based metabolites between treatments are likely to reflect differential lipid metabolism within the TiME due to immunotherapy (105). The machine learning analyses in this study demonstrated high prediction accuracy for treatment responses, highlighting precise spectral markers for each therapy. This study demonstrates that label-free Raman spectroscopy can sensitively detect early biomolecular changes in tumors. This is advantageous in offering valuable insights for clinical monitoring of immunotherapy responses in cancer patients.

## 4 Raman spectroscopy for drug response and metabolomic monitoring

The past years have seen breakthrough achievements in immunotherapeutic interventions including checkpoint inhibitors, cytokine-based immunotherapy, vaccines, and cell therapy (eg, CAR-T cell, CAR-NK cell and TIL therapy). However, the response to immunotherapeutic treatment has been variable among patients, and only a small percentage of cancer patients benefit from this treatment depending on the histological type of tumor and other host factors. In clinical practice, immunohistochemistry (IHC) typically serves as the initial method for assessing patient biomarkers. However, this approach has several limitations, including variability in assay results, ambiguous positivity thresholds, and instances where patients with low expression levels still show therapeutic benefits. It is also heavily dependent on the pathologist’s judgment and experience (106). For patients suffering from cancer, imaging techniques like FDG-PET scans enhance understanding of metabolic changes during immunotherapy (107). Furthermore, radiolabeling checkpoint inhibitors with radioactive isotopes like 89Z allows for PET imaging to track the biodistribution of these inhibitors (108). Despite their utility, these methods often come with challenges related to cost, time, and the need for specialized personnel (109). As shown by some recent studies, researchers can leverage Raman spectroscopy to assess responses to immunotherapeutic drugs while simultaneously examining cancer cell differentiation (69), drug uptake within cells (110), and patterns of cancer metastasis (111, 112).

**Figure 4:**
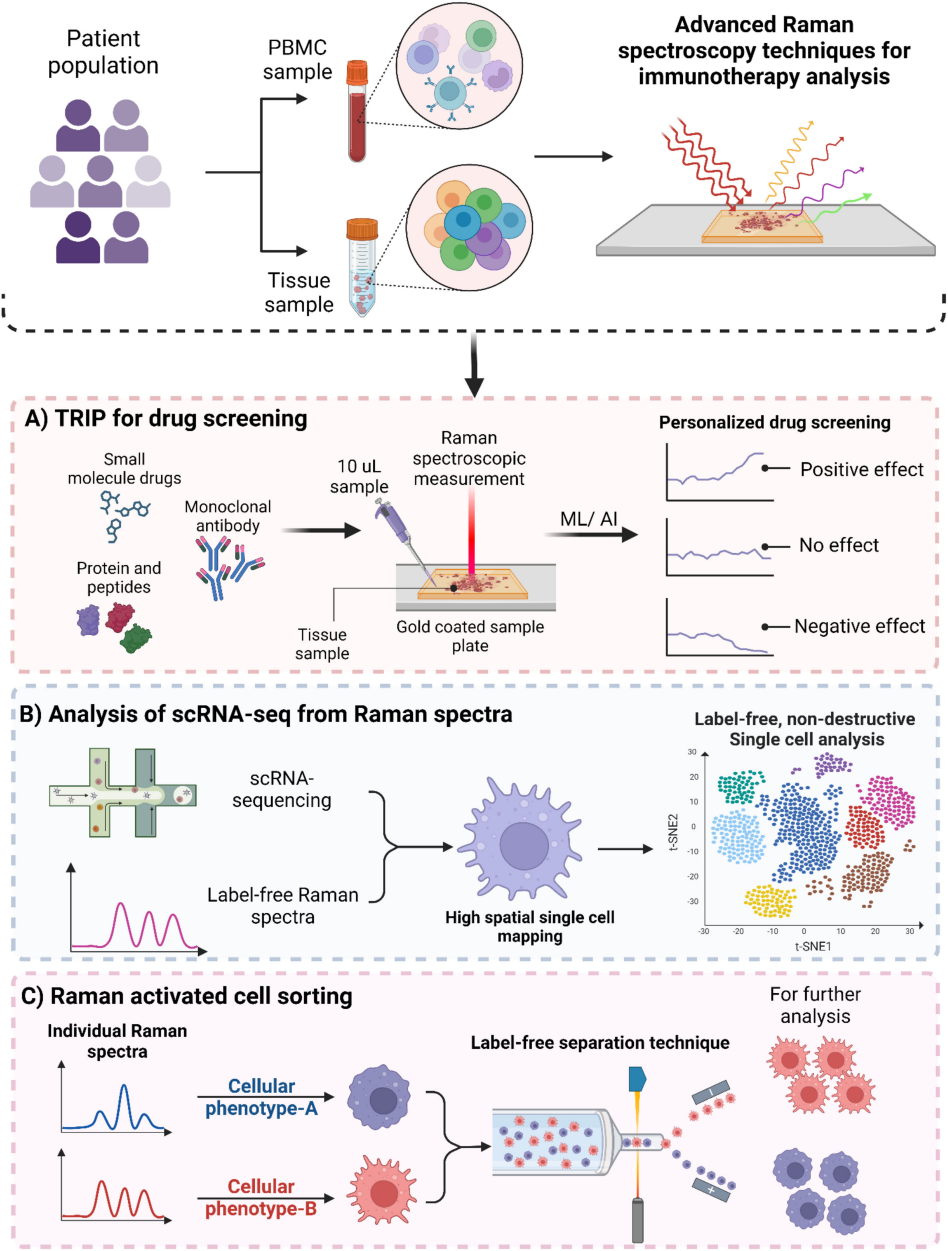
Advanced Raman spectroscopy based techniques for immunotherapy. **(A)** Schematic illustration of Thermostable Raman Interaction Profiling (TRIP) for personalized drug screening. **(B)** Raman spectroscopy based single-cell RNA sequencing for providing high spatial single-cell analysis. **(C)** Raman activated cell sorting (RACS) for label-free cell sorting.

For drug response monitoring, techniques like colorimetric analysis, fluoroimmunoassay, ELISA, and radioimmunoassays are employed, each with distinct advantages and limitations. For instance, the complexity of ELISA protocols often involves multiple incubation and washing steps, making them time-consuming (113). This is especially challenging when working with large sample sizes. Furthermore, the reagents used are costly and can have lot-to-lot variability (114). In immunological studies, researchers commonly use techniques like flow cytometry, ELISA, and confocal microscopy to study the activation, polarization, and plasticity of immune cells along with their cytokine profiles. However, these methods often require the fixation of cells with paraformaldehyde (PFA), the addition of chemical dyes for labeling, and fluorescent tagging with antibodies—either conjugated or unconjugated. Such procedures can be invasive, costly, time consuming and may disrupt biological processes. One notable advancement for label-free drug screening is the Thermostable Raman Interaction Profiling (TRIP) method developed by Altangerel et al. (115). TRIP enables efficient screening of protein-ligand binding at low concentrations and doses under physiologically relevant conditions, as illustrated in **Figure 4A**. TRIP has been successfully applied to eight different protein-ligand systems which demonstrates excellent reproducibility in Raman measurements. The technique requires only a small 10 µL droplet of protein solution on a gold-coated glass slide which dissipates heat from the excitation laser while blocking fluorescent interference. Key applications of TRIP include time-dependent protein-drug binding using 2,4-dinitrophenol (DNP) with transthyretin (TTR), static protein-drug binding involving the streptavidin-biotin complex, and antigen-antibody binding detection with protein A and various antibodies, including those targeting the SARS-CoV-2 spike protein. TRIP is advantageous because of its cost-effectiveness and rapid detection capabilities. This eliminates the need for extensive sample preparation. Future enhancements could enable high-throughput drug screening and real-time monitoring of drug-target interactions, potentially improving drug development processes for complex immunotherapeutic interventions (115).

Single-cell RNA sequencing and other profiling methods allow researchers to study cells in detail, but these techniques destroy the cells during the several processing steps (116). On the other hand, Raman microscopy can analyze the vibrational energy of proteins and metabolites without damaging the cells, achieving a very fine resolution. However, it doesn’t provide genetic information. Raman2RNA (R2R) is a new method that can predict single-cell expression profiles in living cells using label-free hyperspectral Raman images (**Figure 4B**). Either by combining Raman data with single-molecule fluorescence *in situ* hybridization or using advanced machine learning techniques. This kind of approach performed much better than traditional brightfield imaging, with cosine similarities of R2R > 0.85 compared to brightfield < 0.15. When reprogramming mouse fibroblasts into induced pluripotent stem cells, R2R effectively predicted the expression profiles of different cell states. Additionally, while tracking mouse embryonic stem cell differentiation, R2R identified early signs of lineage divergence and development paths (116).

Fluorescence-Activated Cell Sorting (FACS) has been a cornerstone for immunophenotyping and the detailed analysis of immune cell interactions. While FACS bridges the gap between genetic, cellular and population analyses, its reliance on fluorescent probes can interfere with cell metabolism and introduce reliability issues (117) and spectral spillovers (118, 119). Staining the cells with fluorescent dyes also impart cytotoxicity (120), alter the behavior of cells being analyzed (121), and breakdown of dyes which can result in reliability issues. It also limits its application in *in vivo* cell therapies such as stem cell therapy (122) and CAR-T cells (123). In contrast, Raman-Activated Cell Sorting (RACS) presents an exciting alternative. It allows for label-free immunophenotyping by measuring the emitted molecular vibrations of cells as illustrated in **Figure 4C**. RACS integrates multiple technologies to obtain single-cell Raman spectra using different cell-isolation techniques. These methods include operating in a flow environment with microfluidic systems, utilizing Raman tweezers for cellular analysis in solution, and employing Raman Activated Cell Ejection (RACE) for surface-based applications In a study by Wu et al. (124) they developed a novel approach using SERS combined with microfluidic technology to observe real-time interactions between cancer cells and the immune system. This platform is fully automated and integrates optofluidic systems which allows for effective monitoring of these intercellular communications. This integrated system offers several key benefits. Firstly, it facilitates direct on-chip communication between cells. This helps to maintain the bioactivity and concentration of proteins released during interactions, thus closely mimicking the *in vivo* conditions. Secondly, a quantitative SERS immunoassay was employed to evaluate how various drugs influence the secretion patterns of cancer cells and the functionality of immune cells by utilizing an SERS-enhanced 3D barcode immunoassay. Moreover, this automated system significantly minimizes human error and simplifies operational complexity, enhances the reliability of results in drug screening and immunotherapy research.

Amongst most critical applications to date, Raman spectroscopy can probe tumor metabolism in the TiME (125, 126) as growing evidence suggests that the metabolic state of the TiME plays a crucial role in the success of cancer immunotherapy. The TiME can significantly influence the energy consumption and metabolic reprogramming of immune cells, often causing them to become tolerogenic and less effective at eliminating cancer cells. Understanding these metabolic interactions is key to improving immunotherapy outcomes. Unlike mass spectrometry-based single-cell metabolomics, which requires destructive sample preparation (127), label-free Raman spectroscopy can analyze metabolites in living cells and tissues in a non-invasive manner. This makes it well-suited for *in vivo* investigations of tumor metabolism. Recent studies have utilized Raman confocal microscopy combined with ML algorithms to analyze the activation of immune cells such as T cells, B cells, and monocytes (28). For example, Chaudhary et al. employed Raman micro-spectroscopy to identify activated immune cells. Their study included both cell lines and primary cells consisting of purified subgroups of monocytes and lymphocytes, as well as mixed populations of peripheral blood mononuclear cells (PBMCs), all obtained from healthy donors. ML models were designed for cell differentiation and evaluated against flow cytometry data. Spectral signatures of T-cell, B-cell and monocytes before and after activation were also determined using high performance classification models, including spectral fitting to identify spectral biomarkers (28). Importantly, these analyses were conducted alongside traditional methods like flow cytometry and ELISA in both *in vitro* and ex vivo models. The findings indicate that immune cells exhibit unique spectral profiles in response to different stimuli, highlighting the critical roles of both cell type and specific activating signals in shaping their responses. For instance, upon activation, T cells may undergo significant changes in lipid metabolism and protein synthesis, while monocytes might show alterations in cytoskeletal dynamics. These biochemical shifts vary among different immune cell types and are indicative of the complex signaling pathways that govern their activation and differentiation. By examining these spectral changes through Raman spectroscopy, researchers can gain valuable insights into the mechanisms driving immune responses (28). This understanding could pave the way for developing targeted therapeutic strategies aimed at effectively modulating immune function. For example, if specific spectral signatures are associated with effective T cell activation against tumors, therapies could be designed to enhance these pathways for improved cancer treatment outcomes.

## 5 Integrating Raman spectroscopy with -omics approaches and progress towards clinical use

**Figure 5:**
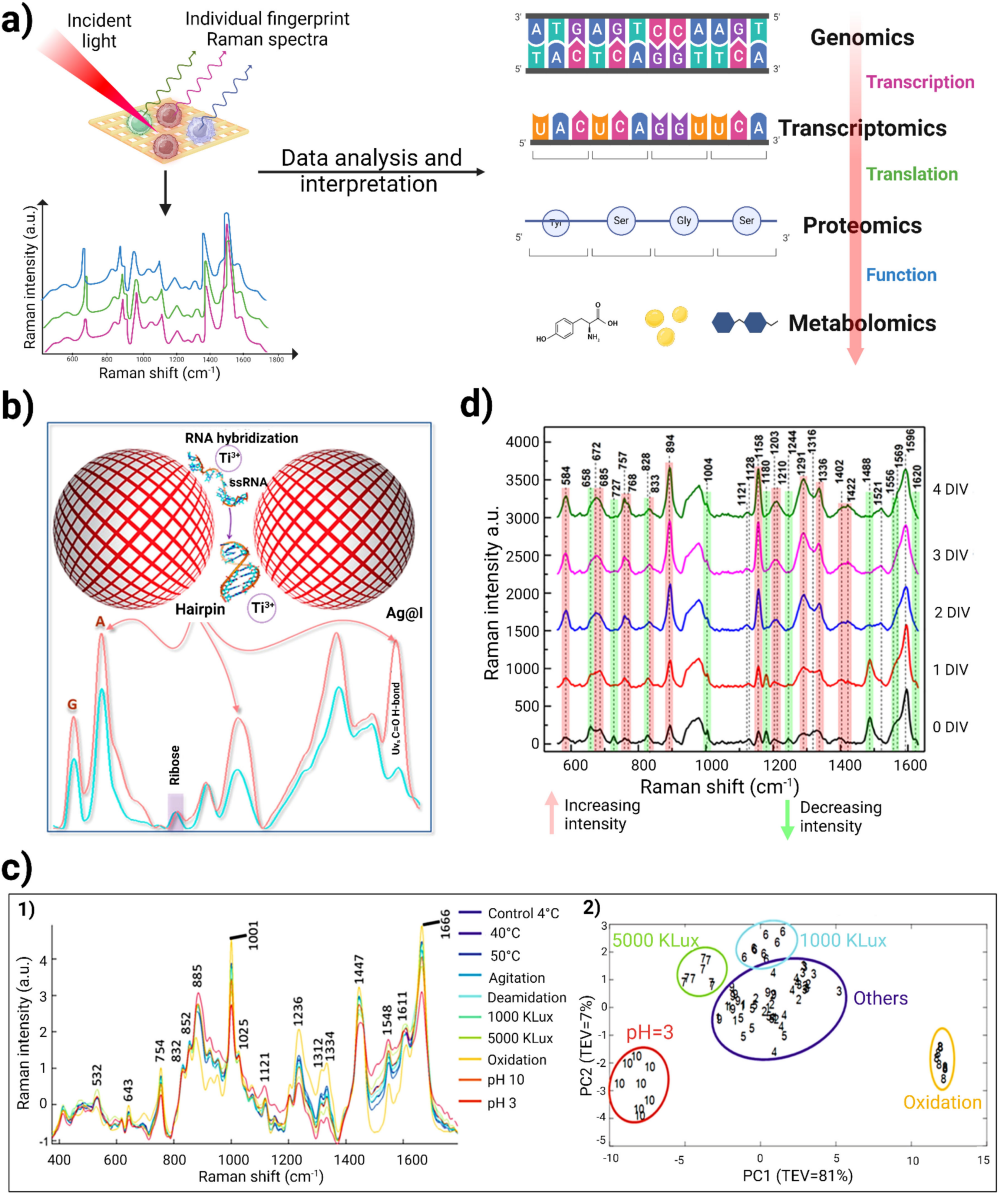
**(A)** Schematic representation of utility of Raman spectroscopy in multi-omics study. When incident light strikes the cells of interest, it generates individual fingerprint Raman spectra. This provides information regarding molecular and chemical composition within cells. Raman spectroscopic data analysis and interpretation using various ML/AI techniques can provide insights for genomics, proteomics, transcriptomics and metabolomics. **(B)** Schematic representation of label-free miRNA identification, using Titanium ions to induce silver nanoparticle “hotspots” to identify RNA sequences of homopolymeric bases and to locate the peak position of each base in the Raman spectrum. Adapted with permission under a Creative Commons CC-BY License from ref (48). **(C)** 1) Raman spectra obtained for 8 different degradation studies of therapeutic monoclonal antibodies was validated against conventional size-exclusion chromatography and peptide mapping. 2) represents the PCA analysis of RS, which can clearly demarcate samples from different degradation clusters (pH 3, oxidation, 5000 kLux·h and 1000 kLux·h) from the control group to allow rapid analysis for therapeutic quality control (132). **(D)** Raman spectra of the DMEM culture medium recorded at various Days *in vitro* (DIV). The red and green lines in the spectra highlight peaks that show increasing and decreasing intensities, respectively. Adapted with permission under a Creative Commons CC-BY License from reference (133).

The “one-size-fits-all” model in immunotherapy often fails to account for individual variations in genetics, environment, and lifestyle, limiting the effectiveness of immunotherapy for many patients (128). Multi-omic approaches that synthesize divergent tumor features such as genomics, transcriptomics, proteomics and metabolomics have significantly advanced the detailed description of heterogeneous tumors and facilitated better understanding of immunotherapy responses (129–131). Integrating multi-dimensional data from various -omics layers remains a significant challenge, and translating these data into precise drug selection for clinical applications has yet to be realized. Additionally, the high costs and labor-intensive nature of genomics, transcriptomics, proteomics, lipidomics, and metabolomics studies require sophisticated analytical and statistical methods. Consequently, these factors have limited the longitudinal capture of events across clinical studies (104, 105). Raman spectroscopy presents a crucial opportunity to harmonize these -omics into a single phenotypic, “Raman-omic” technique. **Figure 5A** illustrates the role of Raman spectroscopy in multi-omics approaches in immunotherapy, to delineate patient heterogeneity, reduce time for analysis, reduce cost associated with those analyses, and harmonize data for better ML/AI analysis by reducing heterogenous data incompatibility. In this section we discuss how Raman spectroscopy can be used to complement and augment genomics, transcriptomics, proteomics, and metabolomics in immunotherapy.

### 5.1 Raman spectroscopy in genomics and transcriptomics

Detecting specific DNA sequences and identifying single-nucleotide polymorphisms (SNPs) are vital for cancer diagnostics and in predicting immunotherapy treatment outcome (134). Next-generation sequencing (NGS) highlights the potential of somatic DNA markers as both independent indicators and novel therapeutic targets (135, 136). Raman spectroscopy has significant potential for studying genomic and transcriptomic alterations. In particular, changes in the vibrational modes of DNA and RNA, including miRNA, can indicate mutations or epigenetic modifications relevant to cancer. Studies have indicated that the activation state of T cells is primarily linked to alterations within DNA rather than proteins (137–139). Chromosomal DNA degradation of activated mature T cells when stimulated via the CD3/T cell receptor complex experience rapid apoptosis. This DNA degradation plays a crucial role in eliminating autoreactive T cells in the thymus (140, 141). In a study by Lee et al., they focused on the Raman spectral analysis of activated mature CD8⁺ T cells and their DNA changes during apoptosis. They noted a decrease in Raman spectral intensities related to DNA, specifically at 768, 1071, and 1463 cm⁻¹. These intensity reductions likely reflect the breakdown of the DNA’s ring structure, signaling its disintegration during apoptosis. Notably, significant changes were observed in the O-P-O region of the DNA backbone (around 780 to 800 cm⁻¹) and in PO₂ (around 1053 to 1087 cm⁻¹). This suggests a correlation with internucleosomal DNA cleavage progression. The differences in Raman spectra between resting and activated mature CD8⁺ T cells were analyzed using PCA which revealed a clear discrimination of DNA from activated T cells compared to resting T cells. Thus, this study infers that the decreased Raman intensities in activated mature CD8⁺ T cell DNA are indicative of apoptosis, highlighting the utility of label-free Raman spectroscopy as a tool for assessing the activation status of these immune cell (142).

In parallel, Li et al. developed a unique detection method for capturing SERS signal from unlabeled RNA without hampering its structural integrity. They utilized titanium ions as an aggregating agent along with silver nanoparticles. This formed electromagnetic “hot-spots” for non-destructive and label-free single molecule detection of miRNA molecules. Unlike traditional metal cation aggregators (like Al³⁺ and Mg²⁺), the acidic titanium ions helped stabilize RNA molecules. The researchers conducted SERS analysis on homopolymeric sequences of the four RNA bases (A, G, C, and U) and examined the secondary hairpin structure (**Figure 5B**). The ribose peak at 959 cm⁻¹ was used for normalization, revealing distinct peak positions for each base: A at 731 cm⁻¹, G at 665 cm⁻¹, C at 789 cm⁻¹, and U at 795 cm⁻¹. To check the robustness of their system, they designed RNA sequences of IL10 and 1HP3 which contained the same bases but in a different sequential manner. A peak at 1446 cm⁻¹ corresponded to U vibrations in AU base pairs, while increases in peak intensities at 1314 cm⁻¹ (G in GC pairs) and 1635 cm⁻¹ (C in GC pairs) indicated complementary pairing. This label-free detection method for miRNA demonstrated a high signal-to-noise ratio with remarkable sensitivity while preserving the original structure of miRNA. This research reduces the analysis cost of miRNA characterization as well as supporting the development of miRNA therapeutics in the future (48).

### 5.2 Raman spectroscopy in proteomics and peptidomics

In the context of cancer diagnosis and new therapeutic development, proteomics plays a valuable role for identifying biomarkers. By analyzing proteins expressed in cancerous tissues compared to healthy tissues, researchers can discern proteins that are uniquely or differentially expressed in either state. Label-free Raman spectroscopy can characterize proteins and their conformational states, providing insights into their roles in cancer. Uzunbajakava et al. demonstrated the first successful use of nonresonant Raman imaging to analyze protein distribution in cells. This study compared Raman images of two cell types: peripheral blood lymphocytes (PBLs) and lens epithelial cells (LECs). The Raman images revealed distinct differences in protein distribution within the nuclei of PBLs and LECs, with clear contrasts in protein intensity visible in the PBL nucleus (near 3000 cm⁻¹) (143). Raman scattering can also be utilized to study the α and β-sheets conformations and changes in proteins. Rygula et al. explored the secondary structures of 26 different proteins (including hemoglobin (Hb), cytochrome c, peroxidase, albumin, collagens, lectins, glucose oxidase, proteinase, ubiquitin, and heme protein) using Raman spectroscopy by analyzing their Amide I and III vibrations, which reveal the ratios of α-helices and β-sheets (144). This research suggests that proteoforms may each have their own vibrational fingerprint. Therefore, even when specific binders are unavailable to discern, eg, post-translationally-modified proteins, Raman can prove specific information about modifications or changes in secondary or tertiary structure.

Peptidomics can also benefit from Raman spectroscopy. For example Raman spectroscopy can help in understanding the roles of specific peptides involved in tumor cell signaling and immune responses (145). Raman spectroscopy has also emerged as a promising tool for detecting post-translational modifications (PTMs) and assessing degradation in monoclonal antibody (mAb) therapeutics (132). These modifications, which occur after protein synthesis, can significantly impact the structure and properties of antibodies, leading to issues like aggregation and fragmentation. PTMs are classified based on the modified amino acids or the enzymes involved, with common modifications including phosphorylation, glycation, acylation, alkylation, glycosylation, deamidation, and oxidation. This is particularly important in mABs, where structural changes can result in unwanted immune reactions (146), decreased effectiveness (147), and material loss during production (148). Monoclonal antibodies are especially vulnerable to aggregation and fragmentation due to various processing conditions with soluble mAB aggregates posing a significant risk for triggering unwanted immune responses (149). A label-free and high throughput Raman spectroscopy can aid in identifying these PTMs in real-time. Due to rapid spectral data collection, little to no sample preparation, and without any interference due to water, Raman spectroscopy emerges as an outstanding candidate for real-time Process Analytical Technology analysis in biotherapeutic production (150). For instance, McAvan et al. studied the effectiveness of label-free RS in detecting PTMs in IgG4 mAbs under various degradation conditions, such as changes in pH (3 and 10), temperature (4, 40, and 50°C), light stresses (1000 and 5000 kLux·h), and agitation. By integrating principal component analysis (PCA) with RS and circular dichroism (CD) spectroscopy, they differentiated mABs based on their PTMs and degradation states. **Figure 5C–1** represents Raman spectra which were obtained for 8 different degradation data. Notably, spectral peaks at 1666 cm⁻¹ and 532 cm⁻¹ remained stable which indicates that β-sheet and disulfide bonds were largely unaffected by these conditions. However, significant changes were observed in the amide III region (1312 to 1334 cm⁻¹), suggesting alterations in the protein’s tertiary structure linked to the degradation conditions. Additionally, RS detected shifts at 885, 1121, and 1450 cm⁻¹ associated with tryptophan and other molecular components, showing that both tryptophan and C−H vibrations increased in wavenumber with larger aggregates. Conversely, the C−N backbone exhibited a decrease in wavenumber as aggregation increased. This research highlights the potential of RS for monitoring PTMs in mAb which were subjected to various forced degradation conditions. The PCA analysis revealed that the data with identical conditions group together. This indicated that the data is consistent and reproducible. Notably, the samples that form distinct clusters apart from the control group include those subjected to oxidation, pH 3, and light exposure at 5000 kLux·h and 1000 kLux·h which is represented in **Figure 5C–2** (132). Furthermore, Zhang et al. used a label-free RS approach along with SVM and PCA model for quantitative prediction of protein aggregation in Antibody Drug Conjugates. Additionally, they have also investigated the impact of temperature and humidity (40°C/75% RH/1 month) on aggregation of proteins that mimics long-term storage conditions (151). These studies suggest that label-free Raman spectroscopy can be used to monitor real-time PTMs during biotherapeutic production.

### 5.3 Raman spectroscopy in metabolomics

Immunometabolomics has become a vital area of study by providing detailed insights into the metabolic interactions within the TiME. The transfer of metabolites between cancer cells and nearby immune cells can shape immune responses, indicating that these metabolic exchanges are key to both immune surveillance and evasion. Research is focused on understanding the vital contribution of metabolic communication between these cells, particularly how tumor metabolism contributes to immune evasion and resistance to immunotherapy (152). Tumor metabolism leads to the buildup of metabolites such as lipids, carbohydrates etc. that regulate immune responses within the TiME (153). These metabolites not only serve as signals but also interfere with the development of immune cells such as CAFs, T-cells and macrophages (154–156). There is an urgent need for new techniques that allow for single-cell metabolic interaction analysis in a quick and cost-effective way. To overcome these hurdles, researchers have utilized Raman spectroscopy for understanding these intricate immune-cell metabolic cross talks. For example, Shalabaeva et al. used a time-resolved method for metabolite tracking in cell culture using label-free SERS, allowing simultaneous analysis of multiple molecules without any sample processing. The method used Ag nanostructures integrated in cell culture medium in a four day study involving NIH/3T3 cells, with Raman spectra collected from media. The analysis of specific peaks revealed temporal changes in metabolic components such as L-tyrosine, L-tryptophan, glycine, L-phenylalanine, L-histidine, and proteins from fetal bovine serum (FBS), as seen in **Figure 5D**. The observed trends for L-tyrosine and its degradation products- acetoacetate and fumarate signified the consumption of L-tyrosine and simultaneously the production of its breakdown products. The decreasing intensity of certain peaks likely indicates exhaustion of these cell medium components over time. This method was also applied to analyze LPS-driven differentiation of Raw 264.7 macrophage cells. Analysis of the Raman spectra collected over a 24hr period reflected macrophage transition from quiescent to an activated pro-inflammatory state. This research indicates that label-free SERS could identify different metabolites at various time points, thereby providing insights into the immune cell states (133).

In cancer metabolomics, lipid metabolism plays a crucial role in cancer development, progression and also influences tumor growth mechanisms, including support for metastasis, ferroptosis-mediated cell death, and interactions between tumor and immune cells (157). Abnormal lipid levels and disrupted metabolic pathways contribute to cancer growth, metastasis, and treatment resistance. As Raman vibrational peaks are exceptionally sensitive for observing lipid content, Raman spectroscopy is increasingly applied for lipidomic analysis across a wide range of cancers (157–159). Lipid droplets (LDs) are dynamic organelles primarily involved in lipid storage and metabolism, and dictating cellular energy balance and signaling. Their significance in cancer biology has garnered attention, particularly regarding resistance to chemotherapy, their interactions with immune cells within the TiME, and implications for immunotherapy (160). It was found that a significantly higher quantity of lipid droplets was present in high-grade glioblastoma and colorectal cancer when compared with low-grade cancers and normal tissues (161). Ben et al. utilized Multiplex coherent anti-Stokes Raman scattering (MCARS), a label-free technique, to detect lipid droplets in colon cancer cell lines expressing the neurotrophin receptor TrkB. The overexpression of TrkB subsequently activates the PI3K/Akt signaling pathway and phosphorylation of Akt (P-Akt), leading to lipid droplet formation in cells. The MCARS technique focused on the 2500–3200 cm−1 spectral range, where the CH2 (2850 cm−1) and CH3 (2930 cm−1) vibrational signatures are primarily associated with lipid and protein contents respectively. MCARS images of cells generated from signal integration of CH2 stretching modes allowed researchers to discriminate between lipid accumulation in the endoplasmic reticulum and the formation of cytoplasmic lipid droplets. This approach tracked the changes in lipid metabolism in both TrkB high-expressing HT29 cells and low-expressing HEK293 cells following treatment with brain-derived neurotrophic factor (BDNF), demonstrating that BDNF-induced TrkB activation leads to lipid droplet formation in HT29 cells. Thus, with MCARS along with data processing, researchers were able to a) detect cancerous cells, b) assess the tumor progression, and c) predict the resistivity of cancer cells by analyzing the content of cytoplasmic lipid droplets (162).

### 5.4 Translational potential of Raman spectroscopy in cancer diagnosis and treatment

Raman spectroscopy is increasingly recognized for its clinical utility in cancer diagnosis and therapy (163, 164). One notable application of label-free Raman spectroscopy is intraoperative margin assessment of brain tumors, particularly glioblastomas. Studies have shown that Raman spectroscopy can differentiate between tumor and healthy brain tissue in real-time during surgical procedures, potentially improving surgical outcomes by ensuring complete tumor resection while preserving surrounding healthy tissue. Jermyn et al. utilized a handheld Raman spectroscopy probe, without any labeling, for real-time detection of cancer cells in human brain tissue during surgery. This technique achieved a sensitivity of 93% and specificity of 91%, effectively distinguishing between normal brain tissue, dense cancer, and cancer-invaded areas. The probe illuminated a 0.5-mm tissue area, sampling up to 1 mm deep in just 0.2 seconds, integrating seamlessly into neurosurgical workflows. The spectra covered a range of shifts from 381 to 1653 cm−1. The Raman spectra revealed distinct differences in lipid bands and nucleic acid content between cancerous and normal tissues. Specifically, variations at 700 cm−1 and 1142 cm−1 indicated changes in cholesterol and phospholipids, while increased bands from 1540 to 1645 cm−1 suggested higher nucleic acid levels in cancerous tissues. With ML analysis, they were able to classify the samples with an overall accuracy of 92% (107). This portable Raman technology enhances intraoperative decision-making by providing quick, reliable identification of invasive cancer, minimizing residual tumor volume and improving patient survival outcomes. Raman spectroscopy has also now been used for real time cancer cell differentiation and diagnosis in oral cancer (165, 166), gastric cancer (167), and skin cancer (168).

Furthermore, Raman spectroscopy is gaining momentum as a non-invasive diagnostic tool in oncology-based clinical trials (**Table 1**). In a recent clinical investigation by Wang et al., serum samples from 729 patients diagnosed with either prostate cancer (PC) or benign prostatic hyperplasia (BPH) were analyzed. The researchers utilized SERS combined with an AI model based on convolutional neural networks (CNN) for diagnostic purposes. Their findings indicate an accuracy of ~85% for distinguishing between PC and BPHBy integrating patient age and prostate-specific antigen (PSA) levels into their multimodal CNN approach, the classification accuracy improved significantly to over 88% (169). Encouraged by these results, the researchers have initiated a clinical trial to explore this diagnostic technique, registered under NCT05854940 (170).

**Table 1:** Current clinical trials of Raman spectroscopy for cancer diagnosis and treatment.

Sr. no	NCT number	Study title	Conditions	Interventions	Brief summary
1	NCT04162431	DOLPHIN-VIVO: Diagnosis Of LymPHoma IN Vivo (Ex Vivo Phase)	Lymphoma; Head and Neck Cancer	Combined FNA/Raman spectroscopy needle probe	Study for the use of Raman spectroscopy for non-invasive analysis of lymph node tissue (*x-vivo* and *in-vivo*) for providing immediate diagnostic results without laboratory delays. It aims to streamline the biopsy process by integrating fine needle aspiration during routine procedures, maintaining clinical standards.
2	NCT05010369	DOLPHIN-VIVO: Diagnosis of LymPHoma in Vivo (*In Vivo* Phase)			
3	NCT06384924	Raman Spectroscopy and Skin Cancer	Skin Cancer; Basal Cell Carcinoma; Squamous Cell Carcinoma	Handheld Raman Spectroscopy probe	Retrospective trial investigating the effectiveness of Raman Spectroscopy in assessing skin cancer tumor size and spread using a handheld probe that gently contacts the skin with laser light. This method aims to enhance diagnostic accuracy and efficiency.
4	NCT06394050	Label-free Raman Spectroscopy for Discrimination Between Breast Cancer Tumor and Adjacent Tissues After Neoadjuvant Treatment	Breast cancer	Label-free Raman spectroscopy based diagnosis	This trial aims to utilize label-free Raman spectroscopy to distinguish between cancerous cells and adjacent tissues in breast cancer patients’ post-treatment.
5	NCT04817449	Spectroscopy in Ovarian Cancer	Ovarian Cancer; Ovarian Neoplasms	Raman spectroscopy	This trial investigates the utility of label-free RS for early detection of ovarian cancer, by analyzing blood plasma (from ovarian cancer patients) and fibrotic tissue (post-chemotherapy) with label-free RS to identify active cancer.
6	NCT04869618	Validation of an Artificial Intelligence System Based on Raman Spectroscopy for Diagnosis of Gastric Premalignant Lesions and Early Gastric Cancer	Gastric Intestinal Metaplasia; Gastric Cancer	AI and Raman spectroscopy-based device (SPECTRA IMDx)	Study for using Raman spectroscopy based artificial intelligence system (SPECTRA IMDx) for early detection and treatment of gastric premalignant lesions and early gastric cancer (EGC).
7	NCT05854940	Multicenter, Prospective Clinical Study of the Serum Raman Spectroscopy Intelligent System for the Diagnosis of Prostate Cancer	Prostate Cancer	Serum Raman spectroscopy intelligent diagnostic system	Trial for validating the effectiveness of RS at screening prostate cancer by detecting prostate-specific antigen (PSA)focusing on early prostate cancer diagnosis.
8	NCT05995990	Raman Spectroscopy for Liver Tumours Following Liver Surgery	Colorectal Cancer Metastatic	Raman Spectrometry	Trial utilizing both RS and multivariate spectral analysis to develop a quick and reliable method for evaluating tissue sections for residual tumors in liver samples after surgery.

In another example, label-free RS has been used for diagnosis and staging of diffuse large B-cell lymphoma (DLBCL) and chronic lymphocytic leukemia (CLL) (171, 172). Label-free Raman spectroscopy (RS) has emerged as a valuable tool for diagnosing and staging diffuse large B-cell lymphoma (DLBCL) and chronic lymphocytic leukemia (CLL). In a study conducted by Chen et al. (2022), label-free SERS spectra were obtained from 47 healthy controls and 53 DLBCL patients. AgNPs was used as a substrate for SERS analysis. Their analysis revealed that DLBCL samples exhibited higher spectral intensities at 725, 1093, 1329, 1371, and 1444 cm⁻¹, indicating the presence of biomolecules such as hypoxanthine, adenine, thymine, collagen, and phospholipids. While lower intensities were observed at 493, 636, 888, 1003, 1133, 1580, and 1652 cm⁻¹ which relate to ergothioneine, uric acid, tyrosine, lactose, phenylalanine, acetoacetate, amide I, and alpha-helix. They also found distinct spectral variations between early-stage (I and II) and late-stage (III and IV) DLBCL. To analyze the complex SERS data effectively, multivariate techniques were employed. The k-nearest neighbors (kNN) model demonstrated better results in both diagnosing and staging DLBCL with an accuracy of 87.3%, sensitivity of 0.921 and specificity rates of 0.809 for diagnosis (171). In another patient-centric study, Bai et al. explored the potential of RS to create a blood test for the noninvasive detection of DLBCL and CLL through biomarker analysis. They examined blood plasma samples from 33 DLBCL patients, 39 CLL patients, and 30 healthy individuals. Their analysis revealed that the intensity at 1445 cm⁻¹, associated with collagen and lipids, was notably higher in DLBCL samples. Conversely, the intensity at 1655 cm⁻¹, linked to proteins and alpha-helix structures decreased in CLL samples while increasing in DLBCL samples. By combining RS with orthogonal partial least squares discriminant analysis (OPLS-DA), the researchers were able to differentiate the blood plasma of CLL and DLBCL patients from that of healthy donors. This integrated approach achieved sensitivity rates of 92.86% for CLL and 80% for DLBCL along with specificity rates of 100% and 92.31%, respectively (172). To further this research, various ongoing clinical trials are investigating both ex vivo and *in vivo* diagnostic methods for lymphoma detection. These trials highlight the current clinical need in cancer diagnostic approaches, especially in cancer immunotherapy. With the advancement of ML and AI technology, integrating RS in biomarker prediction as a diagnostic tool can be crucial for a personalized approach in immunotherapy. This will help solve many current limitations which are present in immunotherapeutic treatment.

## 6 Future directions in label-free assays to develop personalized therapeutic approaches

Label-free Raman spectroscopy in cancer diagnosis and immunotherapy is poised to revolutionize the landscape of oncological care. As a non-invasive diagnostic tool, label-free Raman offers advantages in terms specificity and throughput, enabling the detection of molecular signatures associated with various cancers directly from biofluids such as blood, urine, and saliva (173), distinguishing various tissue types, and detecting pathological alterations across a multitude of diseases. Preclinical, translational, and clinical *in vivo* applications have significantly enhanced Raman spectroscopy’s role in bridging crucial knowledge gaps, especially in the complex analysis of whole-tissue to accurately describe tumor microenvironments. However, several challenges persist in utilizing Raman spectroscopy as a standalone multi-omic test or as a complementary tool to existing multi-omics. Achieving the ambitious goal of entirely label-free assays that are low-cost and high-throughput is essential for accelerating clinical patient studies.

One important step to advancing Raman application for cancers is increasing utilization of formalin-fixed paraffin-embedded (FFPE) specimens, where currently-described studies predominantly concentrate on fresh or frozen tissue samples. FFPE is the conventional method used for the preservation and storage of tissue, especially tumor sampling that is a very small size such as in melanoma or biopsies of metastatic tissues. Due to the dominant vibrational signal native in paraffin, deconvoluting the relatively weak signature of the tissue spectra from paraffin spectra remains a persistent challenge. Robust suppression of the background signal from the paraffin, either through chemical dewaxing demonstrated by Ning et al. and Gaifulina et al (174, 175), digital processing as shown by Tfayli et al. and Ibrahim et al. (176, 177), or vibrational mode-suppressing SERS devices as shown by Kurouski et al. (178), can greatly increase the possible patient data banks available to process and construct the necessary library for the integration of Raman into multi-omic studies. A notable study by Lewis et al. exemplifies this potential by utilizing label-free Raman spectroscopy to compare findings with immunohistochemistry (IHC). They generated Raman spectral maps from FFPE colonic tissues obtained from healthy individuals and used principal components analysis (PCA) to validate their findings against several IHC markers. Their results demonstrated the ability to differentiate mucin based on glycosylation patterns, identify nuclear regions through DNA content analysis, and categorize various tissues according to their amino acid compositions. Their results confirm excellent correlation between the IHC markers and label-free RS. This assures that label-free RS could be utilized in detecting compositional changes, thus eliminating the use of expensive antibodies (179). Ability to access the wealth of banked and stored FFPE could facilitate the next leap in biologic study by greatly expanding available specimens.

A second step for widespread adoption of RS in clinical care, particularly at point of care sites, is efficient sample pre-processing and data post-processing. Clinical integration of sample preprocessing techniques prior would greatly facilitate Raman analysis by eliminating unwanted background and noise. Common sample preparation materials and ubiquitously-present chemical molecules can often obscure and influence relevant functional group vibrational signals. Strategic suppression of non-relevant chemical groups or biological bands either chemically (180) or through Raman-active platforms (181) can greatly improve functional group targeting and better map them to observable biomarker differences. Additional construction of a global spectra library would further assist in signal deconvolution and aid in standardization across samples. Timely tumor profiling will also require rapid integration at subcellular resolution over the entire tissue sample. As such, utilization of higher-throughput Raman systems enabling line- or image-based spectral collection pathways can greatly improve spectral acquisition throughput and capacity. Higher collection bandwidth can aid in the population of a data bank derived from historical samples.

Further advancements in Raman-based tumor investigations necessitate continuous enhancements in the technical performance of spectral acquisition and the resolution of signals. While current SERS devices strategically drive enhancements at the incident light source, signal intensity can further be amplified by additional consideration of enhancements in the scattering wavelength regime. Design of doubly resonant platforms, with the second broader resonance directing Raman scattered light towards the detector, can yield increased spectra intensity and sensitivity. Further, multi-resonant platforms accounting for polarization dependency can enable sample filtering by polarizability. Careful considerations will need to address spectral fidelity associated with fabrication imperfections and hotspot intensities variations across regions. Finally, as tumors and the TiME are most faithfully depicted as a three-dimensional ecosystem, future SERS designs should extend applicability to include all three degrees of spatial freedom. Although confocal RS has been utilized as a 3D molecular contrasting tool (180, 182), similar applications have not yet been applied in SERS-based tumor studies. Potential adoption of suspended or resonantly stratified NPs could provide z-stacking capabilities, while maintaining high-sensitivity. Similarly, considerations will need to be taken to address hotspot uniformity and off-focus signal contributions.

The field of AI and machine learning in Raman spectroscopy data analysis has revolutionized the way we approach real-time data interpretation, particularly in single-cell and multi-omics studies. These LLM models have shown remarkable potential in integrating diverse data types, allowing researchers to simultaneously characterize different cellular processes. However, the journey from laboratory research to clinical application of Raman spectroscopy to immunotherapy principles faces several hurdles. One significant challenge lies in the data acquisition process, which often lacks standardization. Researchers employ varying methods for sample preparation, instrument operation, and data labeling. This leads to inconsistencies across different studies. To address this, the scientific community could benefit from establishing a global, public database for Raman spectroscopy data. This repository would not only store data from laboratories worldwide but also implement standardized normalization and preprocessing techniques, paving the way for more robust AI and ML method development. Another pressing issue is the "black box" nature of many AI models. While these algorithms excel at producing results, the opacity of their decision-making processes can be a stumbling block for clinical adoption. Healthcare professionals understandably hesitate to rely on tools they cannot fully comprehend or explain. Therefore, enhancing the transparency and interpretability of these models is crucial for their acceptance in medical settings. Looking ahead, the field of immunotherapy applications using Raman spectroscopy and AI has several promising fields for growth. Multi-center studies should be prioritized to improve data consistency and reliability, as current research often relies on single-center data. Additionally, the development of semi-supervised or unsupervised machine learning models could unlock new possibilities beyond current applications. These advanced models could potentially uncover hidden correlations between various omics data sets, opening doors for innovative hypothesis testing, drug discovery, and personalized medicine approaches in immunotherapy (35). By addressing these challenges and exploring new frontiers, the integration of AI, Raman spectroscopy, and immunotherapy holds promise for advancing our understanding of cellular processes and improving patient outcomes.

These technical advancements are crucial not only for studying therapeutic responses and discovering biomarkers but also for achieving precision immunotherapeutics. Accurate intraoperative diagnosis for complete tumor resection is essential for improving prognosis and determining optimal surgical approaches in multi-modal care settings.Raman spectroscopy has demonstrated the ability to distinguish malignant tissue from healthy tissue in real-time that can facilitate margin assessment and *in vivo* pathologic classification (183, 184). For example, one recent study applied label-free visible resonance Raman spectroscopy to enhance the precision of tumor boundary identification during glioma surgeries, with remarkable sensitivity, specificity, and accuracy rates reaching 100%, 96.3%, and 99.6%, respectively (185). Looking ahead, the integration of label-free Raman spectroscopy into surgical practice holds significant promise for improving cancer surgery outcomes. As this technology matures, it is expected to facilitate real-time assessments of tumor margins during surgical procedures. This will aid surgeons in achieving complete tumor resections. The development of portable Raman analytical techniques and advanced algorithms for data analysis will further enhance the utility of *in-situ* applications. This will make label-free Raman spectroscopy an invaluable tool in the future landscape of oncological surgery.

## 7 Conclusion

Label-free Raman spectroscopy could transform cancer diagnosis and immunotherapy by offering a non-invasive, high-throughput method for detecting molecular signatures in biofluids and tissue specimens. The studies outlined here highlight the myriad of challenges in multifaceted profiling of complex and heterogeneous tumors that can be addressed with technical innovations in Raman spectroscopy to transcend traditional single-omic strategies. The analytical advancements in Raman technologies, encompassing enhanced spectral isolation and refined data processing capabilities, establish it as a crucial instrument for elucidating the intricate mechanisms by which tumors circumvent immune detection—a critical stride towards precision medicine. Coupled with machine learning for real-time data analysis, these techniques position Raman technology as a disruptive tool throughout the continuum of oncological intervention.As techniques for suppressing background signals improve and as the construction of global spectral libraries advances, the accuracy and efficiency of Raman spectroscopy in clinical settings will be enhanced. The potential integration of Raman spectroscopy with existing multi-omics platforms could harmonize diverse datasets, facilitating a more comprehensive characterization of tumors and better predictive biomarker identification. Moreover, the potential for real-time tumor boundary identification during surgeries positions Raman technology at the forefront of precision immunotherapeutics. The ongoing development of portable systems and sophisticated data analysis algorithms promises to further embed label-free Raman spectroscopy within surgical practice, ultimately improving patient outcomes through more precise and informed interventions. By enabling timely, personalized, and precise immunotherapy strategies, this technology could ultimately transform the landscape of oncological care, reducing reliance on a “one size fits all” treatment paradigm and enhancing patient outcomes.
